# Introduction of a new scoring tool to identify clinically stable heart failure patients

**DOI:** 10.1007/s12471-021-01654-8

**Published:** 2022-01-05

**Authors:** A. J. Gingele, L. Brandts, H. P. Brunner-La Rocca, G. Cleuren, C. Knackstedt, J. J. J. Boyne

**Affiliations:** 1 Department of Cardiology, Maastricht University Medical Centre+, Maastricht, The Netherlands; 2 Department of Clinical Epidemiology and Medical Technology Assessment, Maastricht University Medical Centre+, Maastricht, The Netherlands; 3 Department of Patient and Care, Maastricht University Medical Centre+, Maastricht, The Netherlands; 4 Department of Health Services Research, CAPHRI, Maastricht University, Maastricht, The Netherlands

**Keywords:** Heart failure, Primary health care, Mortality, Referral, Consultation

## Abstract

**Introduction:**

Heart failure (HF) poses a burden on specialist care, making referral of clinically stable HF patients to primary care a desirable goal. However, a structured approach to guide patient referral is lacking.

**Methods:**

The Maastricht Instability Score—Heart Failure (MIS-HF) questionnaire was developed to objectively stratify the clinical status of HF patients: patients with a low MIS-HF (0–2 points, indicating a stable clinical condition) were considered for treatment in primary care, whereas high scores (> 2 points) indicated the need for specialised care. The MIS-HF was evaluated in 637 consecutive HF patients presenting between 2015 and 2018 at Maastricht University Medical Centre.

**Results:**

Of the 637 patients, 329 (52%) had a low score and 205 of these 329 (62%) patients were referred to primary care. The remaining 124 (38%) patients remained in secondary care. Of the 308 (48%) patients with a high score (> 2 points), 265 (86%) remained in secondary care and 41 (14%) were referred to primary care. The primary composite endpoint (mortality, cardiac hospital admissions) occurred more frequently in patients with a high compared to those with a low MIS-HF after 1 year of follow-up (29.2% vs 10.9%; odds ratio (OR) 3.36, 95% confidence interval (CI) 2.20–5.14). No significant difference in the composite endpoint (9.8% vs 12.9%; OR 0.73, 95% CI 0.36–1.47) was found between patients with a low MIS-HF treated in primary versus secondary care.

**Conclusion:**

The MIS-HF questionnaire may improve referral policies, as it helps to identify HF patients that can safely be referred to primary care.

**Supplementary Information:**

The online version of this article (10.1007/s12471-021-01654-8) contains supplementary material, which is available to authorized users.

## What’s new?

- The Maastricht Instability Score—Heart Failure questionnaire is a promising and feasible tool that can support physicians in identifying clinically stable heart failure (HF) patients.
- Clinically stable HF patients can safely be referred from secondary to primary care.
- A standardised evaluation of HF patients can improve communication between different care givers and can facilitate collaboration between primary and secondary care.

## Introduction

Heart failure (HF) is a severe clinical syndrome with high prevalence, affecting approximately 1–2% of the general population in Western countries [1]. Health care consumption among HF patients is extensive, mainly due to frequent hospital admissions and outpatient clinic visits. Together, this poses a significant burden on HF specialists [2, 3], making a stronger involvement of primary care desirable.

However, it has been questioned whether primary care can meet the complex demands of HF patients [4]. So far, mixed results have been obtained regarding differences in guideline adherence and hospital re-admission rates between primary and secondary HF care [5–8]. Stable HF patients with mild symptoms may be safely referred to primary care, whereas patients with moderate to severe HF might need more targeted cardiovascular care by HF specialists [8].

Consequently, the Dutch Ministry of Health strongly advocates multidisciplinary and integrated HF care, characterised by intensive collaboration between primary and secondary care [9, 10]. With general practitioners (GPs) as a vital part of multidisciplinary teams, referral of HF patients to primary care could not only increase the accessibility of HF care and lower costs, but may also reduce the burden on secondary care [11]. In turn, instable HF patients should easily regain access to secondary care if required. This multidisciplinary integrated approach was endorsed by the Netherlands Society of Cardiology (NVVC) [12]. However, clinical criteria to define HF patients that are sufficiently stable for referral to primary care are lacking. Therefore, in the absence of guidelines, this decision is based solely on the health care provider’s judgement.

In order to achieve a more objective referral policy, we developed the Maastricht Instability Score—Heart Failure (MIS-HF) questionnaire, a tool enabling the structured evaluation of a patient’s eligibility for referral from secondary to primary care. The questionnaire is based on common HF signs, symptoms and frequently reported clinical complications of HF. The aim of this study was to evaluate the reliability, feasibility and safety of the MIS-HF questionnaire in referral of HF patients from secondary to primary care.

## Methods

### Study procedure

A working group comprising HF cardiologists, GPs, epidemiologists, as well as HF nurses and general practice nurses, was established in Maastricht, the Netherlands. Members were asked to define a set of criteria to assess the clinical condition of individual HF patients. In different meetings, all relevant aspects with respect to referral were gathered in an iterative process. The selection of items was based on the clinical experience of the participating HF professionals as well as current HF guidelines [13]. In total, 33 criteria were identified and translated into a dedicated questionnaire, the MIS-HF questionnaire (Tab. 1). Signs and symptoms of HF, biomarkers, electrocardiographic and imaging characteristics, as well as psychosocial measures, were included. A MIS-HF of 0, 1 or 2 was defined as a stable clinical condition, whereas a score of 3 or higher suggested clinical instability. This particular threshold was chosen quite conservatively in order to increase the specificity of the instrument, preventing referral of clinically unstable patients to primary care. The working group agreed that patients with a low score (total score 0, 1 or 2) could be referred to primary care for further treatment, whereas patients with a high score (total score 3 or higher) should be further treated by the HF specialist. Based on the MIS-HF a recommendation was provided to the treating physician. Importantly, the final decision as to whether to follow up the patient in primary or secondary care was left to the discretion of the treating physician and patient.

**Table 1:** Maastricht Instability Score—Heart Failure questionnaire scoring list

Item	Score
NYHA 1	0
NYHA 2	0
NYHA 3	1
NYHA 4	3
*Dyspnoea*	
No dyspnoea, dyspnoea unaltered/improved	0
Worsening of dyspnoea during exercise	1
Orthopnoea, waking up with dyspnoea (new)	3
*Blood pressure*	
BP < 90/50 mm Hg with symptoms of hypotension	1
BP >140/85 mm Hg	1
*Heart rate*	
Sinus rhythm > 75 beats/min	2
Atrial fibrillation > 100 beats/min	2
Irregular heart rhythm/atrial fibrillation (new)	2
Irregular heart rhythm/atrial fibrillation with symptoms	2
*Weight*	
Increased > 2 kg during 1 week	1
Decreased	1
Decreased with signs of cachexia	2
*Oedema*	
Absent	0
Present	1
*Angina pectoris*	
No/stable CCS ≤ 2	0
Progressive	3
Class 3	2
*Other*	
NT-proBNP increased > 25%	1
NT-proBNP > 400 pmol/ l (> 3383 pg/ml)	1
Potassium < 3.5 or > 5.0 mmol/l	1
Sodium < 135 or > 145 mmol/l	1
Creatinine > 220 µmol/l or increased > 25% or GFR < 30 ml/min	1
Haemoglobin < 6.5 mmol/l; < 10.5 g/dl (new)	2
Haemoglobin < 6.5 mmol/l; < 10.5 g/dl (chronic)	1
Up-titration of HF medication to maximum tolerated doses not achieved	1
Poor compliance with therapy (suspected)	1
Poor social support	1
Signs of depression	1
Hospital admission due to HF (≥ 1 during last 6 months, ≥ 2 during last year)	2
	**Total score:**

*NYHA* New York Heart Association Functional Classification, *BP* blood pressure, *CCS* chronic coronary syndrome, *NT-proBNP* N-terminal pro-brain natriuretic peptide, *GFR* glomerular filtration rate, *HF* heart failure

The MIS-HF was obtained by trained HF nurses in all patients meeting our inclusion criteria (≥ 18 years of age, diagnosed with HF) that visited our HF outpatient clinic. Patients with less than 1 year on cardiac resynchronisation therapy, pre- or post-heart transplant, valvular or ischaemic heart disease with planned or recent surgery, planned (percutaneous) interventions or undergoing palliative care were excluded. In randomly selected cases, two HF nurses independently completed the MIS-HF questionnaire for the same patient to evaluate inter-observer variability.

### Data collection

MIS-HF questionnaires completed at our HF outpatient clinic were collected between September 2015 and September 2018. Clinical data about referral status, mortality and number of HF-related and cardiac non-HF-related hospital admissions were obtained from the hospital information system.

Our study was approved by the local ethics committee of Maastricht University Medical Centre (METC/2020-2363). Informed consent was obtained from all participants during their first outpatient clinic visit. The investigation conforms to the principles outlined in the Declaration of Helsinki [14].

### Endpoints

The primary endpoint of our retrospective cohort study was a composite of all-cause mortality or HF-related or cardiac non-HF-related hospital admissions within 1 year. Secondary endpoints were all-cause mortality, HF-related hospital admissions and cardiac non-HF-related hospital admissions within 1 year.

### Statistical analysis

Descriptive statistics stratified by referral status and MIS-HF were used to describe the population’s baseline characteristics. An independent *t*-test (if data were normally distributed) or Mann-Whitney test (if data were not normally distributed) was used to analyse differences in baseline variables. Inter-rater reliability was calculated using Cohen’s kappa and intra-class correlation coefficients. Internal validity was measured with Cronbach’s alpha. Univariable-adjusted and multivariable-adjusted logistic regression analyses were performed to estimate the association between referral class (referred to a GP with MIS-HF 0–2, referred to a GP with MIS-HF > 2, not referred to a GP with MIS-HF 0–2, and not referred to a GP with MIS-HF > 2) and study endpoints. Multivariable adjusted models were adjusted for age (years, continuous), sex (male/female), chronic obstructive pulmonary disease (COPD; yes/no) and diabetes mellitus (yes/no). The presence of COPD and diabetes mellitus was based on physician diagnosis. Applying a sensitivity analysis, additional adjustments were made for the presence of a pacemaker (yes/no), presence of an implantable cardioverter defibrillator (ICD; yes/no) and use of telemonitoring (yes/no). The associations were presented using the odds ratio (OR), including the 95% confidence interval (95% CI), and the *p*-value. A *p*-value < 0.05 was considered statistically significant. All analyses were performed using SPSS version 15.0.

## Results

Overall, we evaluated 637 elderly HF patients using the MIS-HF questionnaire (Electronic Supplementary Material; Baseline characteristics). Mean age was 74.3 years (SD 12.0), just over half were male (57.3%) and 68.9% were New York Heart Association (NYHA) class II or lower. In total, 52% of the patients had a low MIS-HF and 48% had a high score. Of those with a low score, 62% were referred to primary care, whereas 86% of patients with a high score remained under treatment in secondary care. Patients referred to primary care despite a high MIS-HF (*n* = 41, mean age 79.7 years, SD 8.5) were significantly older than patients treated in secondary care with a high MIS-HF (*n* = 267, mean age 74.0 years, SD 12.1). Patients treated by a cardiologist despite a low MIS-HF (*n* = 124, mean age 71.4 years, SD 12.3) were significant younger, had higher rates of aortic valve stenosis (12.1% vs 6.3%) and were more likely to be treated with a beta blocker (88.7% vs 78.5%) than patients referred to primary care based on their low MIS-HF (*n* = 205, mean age 75.4 years, SD 11.9). No significant differences were observed between those groups in NYHA class, ejection fraction, N‑type pro-brain natriuretic peptide (NT-proBNP) levels or comorbidities (diabetes mellitus, COPD, hypertension).

### Clinical outcomes

**Fig. 1:**
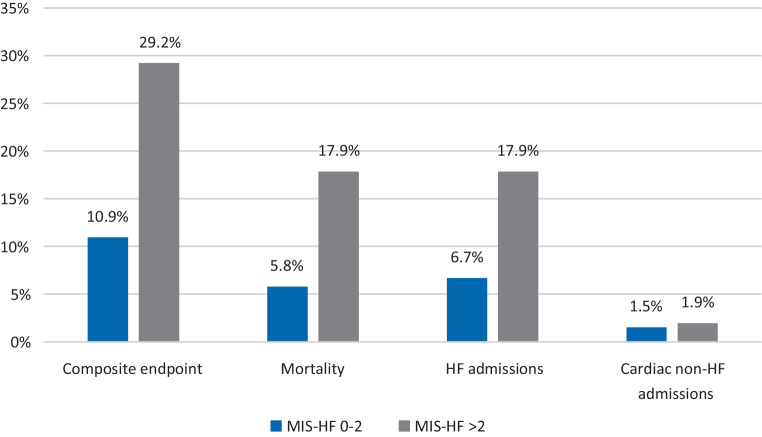
Percentage of patients with low and high scores on the Maastricht Instability Score—Heart Failure questionnaire reaching primary and secondary endpoints. Composite endpoint = all-cause mortality, heart failure (*HF*) admissions and cardiac non-HF admissions

After 1 year of follow-up, the composite primary endpoint occurred more frequently in patients with a high MIS-HF compared to those with a low score (OR 3.36, 95% CI 2.20–5.14). Patients with a high MIS-HF had significantly more HF-related hospital admissions (OR 3.30, 95% CI 1.96–5.55) and higher all-cause mortality (OR 3.55, 95% CI 2.05–6.13) than patients with a low score (Fig. 1). Cardiac non-HF-related hospital admissions were rare and did not differ between the two groups (OR 1.29, 95% CI 0.39–4.26).

In patients with a low MIS-HF (0–2), the composite endpoint and HF-related hospital admissions tended to be higher in secondary compared to primary care, whereas mortality rates were lower. Of note is that these differences were not statistically significant (Tab. 2). After multivariable adjustment for age, sex, COPD and diabetes mellitus, the OR for mortality was lower compared to univariable analysis (OR 0.96, 95% CI 0.34–3.70), whereas the ORs for hospitalisation were unaltered. Similar results were obtained after additional adjustment for ICD, pacemaker or telemonitoring usage. None of those trends was statistically significant.

**Table 2:** Hospital admission, all-cause mortality and composite endpoint in patients with low Maastricht Instability Score—Heart Failure (*MIS-HF*) treated by a general practitioner (*GP*) or cardiologist

Outcome	MIS-HF 0–2 GP (*n* = 205)	MIS-HF 0–2 cardiologist (*n* = 124)	Odds ratio (95% CI)	*p*-value
HF admission: *n* (%)	11 (5.4)	11 (8.9)	0.58 (0.24–1.39)	0.22
Cardiac admission: *n* (%)	3 (1.5)	2 (1.6)	0.91 (0.15–5.50)	0.91
Mortality: *n* (%)	13 (6.3)	6 (4.8)	1.33 (0.49–3.60)	0.57
Composite endpoint: *n* (%)	20 (9.8)	16 (12.9)	0.73 (0.36–1.47)	0.38

Values are presented as number (%). Mortality = all-cause mortality

*95% CI* 95% confidence interval, *HF* heart failure

Of the 329 patients with a low MIS-HF, 205 were referred to primary care. During 1 year of follow-up, 56 patients (27%) revisited the HF outpatient clinic, of whom 45 continued their treatment in secondary care. Median time to first outpatient clinic visit was 168 days (interquartile range = 100–212). Reported reasons for outpatient clinic visits were exacerbation of HF (*n* = 17; 30%) and recent HF hospital admission (*n* = 4; 7%). No medical reason for returning to secondary care could be identified in the remaining cases.

In patients with a high MIS-HF (> 2), no statistically significant differences in the composite endpoint, HF-related hospital admissions or mortality were found between primary and secondary care (Tab. 3). No cardiac admissions were reported in the primary care group; therefore no OR could be calculated.

**Table 3:** Hospital admission, all-cause mortality and composite endpoint in patients with a high Maastricht Instability Score—Heart Failure (*MIS-HF*) treated by a general practitioner (*GP*) or cardiologist

Outcome	MIS-HF > 2 GP (*n* = 41)	MIS-HF > 2 cardiologist (*n* = 267)	Odds ratio (95% CI)	*p*-value
HF admission: *n* (%)	4 (9.8)	51 (19.1)	0.46 (0.16–1.34)	0.16
Cardiac admission: *n* (%)	0 (0.0)	6 (2.2)	–	0.99
Mortality: *n* (%)	7 (17.1)	48 (18.0)	0.94 (0.39–2.25)	0.90
Composite endpoint: *n* (%)	9 (22.0)	81 (30.3)	0.65 (0.30–1.42)	0.28

Values are presented as number (%). Mortality = all-cause mortality

*HF* heart failure, *95% CI* 95% confidence interval

### Reliability

In 173 patients, the MIS-HF was independently evaluated by two different care givers. Cohen’s kappa for classifying a patient as suitable (MIS-HF 0–2) or not suitable (MIS-HF > 2) for referral to primary care was 0.65. The intra-class correlation coefficient for exact MIS-HF scores was 0.92; Cronbach’s alpha reliability coefficient was 0.45.

## Discussion

Our study aimed to develop a tool facilitating a reliable and objective clinical evaluation of HF patients as well as standardisation of referral policies from secondary to primary care. Patients with a low MIS-HF had a better prognosis than those with a high MIS-HF after 1 year of follow-up, both in primary as well as in secondary care. Furthermore, no differences in clinical endpoints were observed between patients with a low MIS-HF treated in primary care and those remaining in secondary care.

In the NorthStar study, no differences in the primary composite endpoint (death or cardiovascular admission), mortality, HF-related or cardiac non-HF-related hospital admissions were found between stable HF patients treated by a GP or cardiologist [8]. This is in line with our results, which showed no statistically significant differences in prognosis between patients with a low MIS-HF referred to primary care and patients with a low MIS-HF remaining in secondary care.

In the Netherlands, the Dutch GP guidelines provide evidence-based instructions for HF management in primary care, comparable to the ESC HF guidelines [13, 15]. Adherence to those Dutch GP guidelines is high in the Netherlands [16]. This was confirmed by the COACH-2 study, which found no significant difference in HF guideline adherence between primary and secondary care in the Netherlands [7]. This might explain why no statistically significant difference in the prognosis of stable HF patients between primary and secondary care was observed in the present study. However, a trend towards more HF hospital admissions could be seen in the low MIS-HF group remaining in secondary care compared to patients with a low MIS-HF in primary care. These results are in line with the observations of Jong et al. in a Canadian HF population [17]. It might be that HF specialists tend to be more alert to signs of (potential) clinical deterioration of HF patients than generalists, resulting in higher admission and re-admission rates. In addition, patients with a low MIS-HF in primary care were significantly older and, in the case of deterioration, may have refused to be hospitalised [18]. This age difference might also explain why the trend towards higher mortality in the primary care group disappeared after multivariable adjustment for age, sex, COPD and diabetes mellitus.

The WHICH? trial compared home-based to clinic-based patient management in a moderate- to high-risk HF population [19]. No differences in mortality, HF-related or cardiac-related hospital admission were observed between the two approaches. This corresponds to our results showing that patients with a high MIS-HF in primary care had similar outcomes to patients in secondary care. HF hospitalisation rates tended to be even higher in the secondary care group, perhaps due to more advanced symptoms in that group (53% of patients in NYHA classes III–IV, compared to 42% in primary care). However, our results have to be interpreted with caution given the small number of patients referred to primary care despite having a high MIS-HF, with far fewer events than patients treated by a HF specialist based on their MIS-HF.

Most items included in the MIS-HF questionnaire can be collected during medical history taking and physical examination. This is comparable to the approach of Kelder at al., who successfully developed a diagnostic rule for patients suspected of new-onset HF [20]. Except for NT-proBNP levels, all items in their tool were based on medical history and physical examination. These results highlight the importance of signs and symptoms in the evaluation of HF patients.

In the absence of a gold standard, the definition of ‘correctly referred’ patients is purely based on HF specialists’ professional judgement. Defining items to develop the MIS-HF questionnaire was also based on expert opinion using a broad range of signs, symptoms, biomarkers, electrocardiographic and imaging characteristics, as well as psychosocial measures, covering the phenotypical and pathophysiological heterogeneity of the HF syndrome [21, 22]. However, many of these factors are known to be related to poor outcome and therefore related to clinical instability of patients. Very high inter-rater reliability indicates substantial agreement between care givers using the MIS-HF questionnaire. This is an important prerequisite for the consistent use of this tool in clinical practice. As expected, the internal consistency of the MIS-HF was low because signs and symptoms vary greatly between HF patients [23, 24].

Overall, introducing the MIS-HF questionnaire in the clinical management of HF patients is a promising step towards a more objective referral policy in HF care. Patients can be evaluated in a standardised, reliable manner, which may improve communication between different care givers and may facilitate collaboration between primary and secondary care. This is in line with the recommendations of the Dutch NVVC Connect Heart Failure programme, which aims to promote multidisciplinary HF care [25]. Through improved cooperation, GPs, HF specialists and other health care professionals can enhance the quality of HF care, making it more accessible and affordable. Recently, the newest version of the Dutch GP guidelines on HF was published. [15]. In contrast to previous editions, it is now recommended that HF therapy is initiated by HF specialists instead of GPs. Therefore, the number of HF patients (unnecessarily) treated in secondary care might increase further, highlighting the importance of tools like the MIS-HF questionnaire. However, prospective, ideally randomised studies are necessary to clarify the role of the questionnaire in multidisciplinary HF care.

### Limitations

After inclusion, 27% of patients initially referred to primary care visited a HF specialist during follow-up and 22% were subsequently treated by a HF specialist. Clinical deterioration was the most reported cause. However, in more than half of the patients, no distinct cause could retrospectively be identified. Most likely, patients decided to continue their treatment in secondary care after their first GP visit. Due to cross-over, differences in outcome between primary and secondary care group might be underestimated.

The MIS-HF questionnaire was administered to all patients visiting our HF outpatient clinic. Subsequently, a suggestion for referral was made to the treating physician. Due to this non-randomised design, it is not possible to estimate whether referral using the MIS-HF questionnaire is superior to standard of care.

After inclusion, 26% of all patients (*n* = 165) were not referred in line with their MIS-HF due to the HF specialist’s and/or patient’s preference. Most of these patients continued their treatment in secondary care (*n* = 124). This may be due to the fact that we included a HF population that had already been treated by a HF specialist for a long period of time, even when patients were in a stable clinical condition. Therefore, patients were used to secondary care, and both patients and care givers may have doubted the GPs’ ability to provide equivalent HF therapy. However, our present results do not support this doubt. Patients referred in line with their MIS-HF had significantly higher NYHA classifications and NT-proBNP levels at baseline compared to patients not referred in line with their MIS-HF.

Laboratory values were collected only if clinically indicated, based on current HF guidelines. This approach generated missing values, which might have resulted in false low MIS-HF. Therefore, the discriminatory power of the MIS-HF tool could be underestimated. On the other hand, this practical approach makes the MIS-HF questionnaire a feasible tool that can be directly implemented in clinical practice.

Finally, we did not test the MIS-HF for referral from primary to secondary care, which may be an additional application for this clinical score.

## Conclusion

The MIS-HF questionnaire appears to be a promising and feasible tool that can support physicians in identifying clinically stable HF patients. Subsequently, these patients can safely be referred from secondary to primary care. Nonetheless, randomised controlled clinical trials are needed to prove the clinical usefulness of the MIS-HF before the broad implementation of MIS-HF-guided patient referral can be advocated.

## Supplementary Information

Baseline characteristics

## References

[1] Savarese G, Lund LH (2017). Global public health burden of heart failure. Card Fail Rev.

[2] Fang J, Mensah GA, Croft JB, Keenan NL (2008). Heart failure-related hospitalization in the U.S., 1979 to 2004. J Am Coll Cardiol.

[3] Mosalpuria K, Agarwal SK, Yaemsiri S (2014). Outpatient management of heart failure in the United States, 2006–2008. Tex Heart Inst J.

[4] Lowe J, Candlish P, Henry D, Wlodarcyk J, Fletcher P (2000). Specialist or generalist care? A study of the impact of a&nbsp;selective admitting policy for patients with cardiac failure. Int J Qual Health Care.

[5] Edep ME, Shah NB, Tateo IM, Massie BM (1997). Differences between primary care physicians and cardiologists in management of congestive heart failure: relation to practice guidelines. J Am Coll Cardiol.

[6] Philbin EF, Weil HF, Erb TA, Jenkins PL (1999). Cardiology or primary care for heart failure in the community setting: process of care and clinical outcomes. Chest.

[7] Luttik ML, Jaarsma T, van Geel PP (2014). Long-term follow-up in optimally treated and stable heart failure patients: primary care vs. heart failure clinic. Results of the COACH-2&nbsp;study. Eur J Heart Fail.

[8] Schou M, Gustafsson F, Videbaek L (2013). Extended heart failure clinic follow-up in low-risk patients: a&nbsp;randomized clinical trial (NorthStar). Eur Heart J.

[9] Engelfriet PM, Haeck J, Wittekoek-Ottevanger L, Veerman J, Schellekens W (2009). Zorg voor hartfalen zonder falen. Indicatoren voor toezicht op de hartfalenketen.

[10] Kaljouw M, Wijma S (2020). Samenwerken aan passende zorg: de toekomst is nú. Actieplan voor het behoud van goede en toegankelijke gezondheidszorg.

[11] MacInnes J, Williams L (2018). A review of integrated heart failure care. Prim Health Care Res Dev.

[12] Ansink JM, Burgers JS, Geerders BP, Elsendoorn M, van Laarhoven H, Mosterd A (2015). Landelijke Transmurale Afspraak Hartfalen.

[13] Ponikowski P, Voors AA, Anker SD (2016). 2016 ESC guidelines for the diagnosis and treatment of acute and chronic heart failure: the task force for the diagnosis and treatment of acute and chronic heart failure of the European society of cardiology (ESC). Developed with the special contribution of the heart failure association (HFA) of the ESC. Eur J Heart Fail.

[14] World Medical Association (2013). World medical association declaration of Helsinki: ethical principles for medical research involving human subjects. JAMA.

[15] NHG-Richtlijnen. NHG-Standaard Hartfalen. 2021. https://richtlijnen.nhg.org/standaarden/hartfalen. Accessed: 20.07.2021.

[16] Grol R, Braspenning J, Hulscher M (2010). Implementatie van NHG-Standaarden: succes of probleem?. HUISARTS WETENSCHAP.

[17] Jong P, Gong Y, Liu PP, Austin PC, Lee DS, Tu JV (2003). Care and outcomes of patients newly hospitalized for heart failure in the community treated by cardiologists compared with other specialists. Circulation.

[18] Barry PP, Crescenzi CA, Radovsky L, Kern DC, Steel K (1988). Why elderly patients refuse hospitalization. J Am Geriatr Soc.

[19] Stewart S, Carrington MJ, Marwick TH (2012). Impact of home versus clinic-based management of chronic heart failure: the WHICH? (which heart failure intervention is most cost-effective &amp; consumer friendly in reducing hospital care) multicenter, randomized trial. J Am Coll Cardiol.

[20] Kelder JC, Cramer MJ, van Wijngaarden J (2011). The diagnostic value of physical examination and additional testing in primary care patients with suspected heart failure. Circulation.

[21] Iorio A, Pozzi A, Senni M (2017). Addressing the heterogeneity of heart failure in future randomized trials. Curr Heart Fail Rep.

[22] Shah AM, Solomon SD (2012). Phenotypic and pathophysiological heterogeneity in heart failure with preserved ejection fraction. Eur Heart J.

[23] Albert N, Trochelman K, Li J, Lin S (2010). Signs and symptoms of heart failure: are you asking the right questions?. Am J Crit Care.

[24] Abebe TB, Gebreyohannes EA, Tefera YG, Abegaz TM (2016). Patients with HFpEF and HFrEF have different clinical characteristics but similar prognosis: a&nbsp;retrospective cohort study. BMC Cardiovasc Disord.

[25] NVVC Connect. Connect 2021–2025 Samen Verbeteren wij de zorg voor hartpatiënten. 2021. https://www.nvvc.nl/PDF/Connect/NVVC%20Connect%202021-2025%20def.pdf. Accessed: 20.07.2021

