# Supplementary material for: Introduction of a new scoring tool to identify clinically stable heart failure patients
Source: Neth Heart J. 2022 Jan 5;30(9):402–10. doi: 10.1007/s12471-021-01654-8 (PMC9402836; doi:10.1007/s12471-021-01654-8)
Supplement: Supplementary file 1 — Baseline characteristics [file 12471_2021_1654_MOESM1_ESM.docx]

**Electronic Supplementary Material**

**Baseline characteristics**

| **Variable** | **N** | **Overall** | **N** | **MIS-HF < 2 GP** | **N** | **MIS-HF< 2 cardiologist** | **p-**  **value** | **N** | **MIS-HF > 2 cardiologist** | **N** | **MIS-HF > 2 GP** | **p-value** |
| --- | --- | --- | --- | --- | --- | --- | --- | --- | --- | --- | --- | --- |
| Age | 637 | 74.3 ± 12.0 | 205 | 75.4 ± 11.9 | 124 | 71.4 ± 12.3 | **p=**  **0.004** | 267 | 74.0 ± 12.1 | 41 | 79.7 ± 8.5 | **p=**  **0.004** |
| Gender | 637 |  | 205 |  | 124 |  | p=  0.75 | 267 |  | 41 |  | p=  0.27 |
| Male |  | 365 (57.3) |  | 117 (57.1) |  | 73 (58.9) |  |  | 155 (58.1) |  | 20 (49) |  |
| Female |  | 272 (42.7) |  | 88 (42.9) |  | 51 (41.1) |  |  | 112 (41.9) |  | 21 (51) |  |
| **NYHA** | 637 |  | 205 |  | 124 |  | p=  0.23 | 267 |  | 41 |  | p=  0.09 |
| NYHA I |  | 95 (14.9) |  | 54 (26.3) |  | 17 (13.7) |  |  | 19 (7.1) |  | 5 (12.2) |  |
| NYHA II |  | 344 (54) |  | 122 (59.5) |  | 96 (77.4) |  |  | 107 (40.1) |  | 19 (46.3) |  |
| NYHAIII |  | 189 (29.7) |  | 29 (14.1) |  | 11 (8.9) |  |  | 132 (49.4) |  | 17 (41.5) |  |
| NYHA IV |  | 9 (1.4) |  | 0 (0) |  | 0 (0) |  |  | 9 (3.4) |  | 0 (0) |  |
| **Rhythm last ECG** | 636 |  | 204 |  | 124 |  | p=  0.81 | 267 |  | 41 |  | p=  0.83 |
| Sinus rhythm |  | 326 (51.2) |  | 106 (51.7) |  | 65 (52.4) |  |  | 134 (50.2) |  | 21 (51.1) |  |
| Atrial fibrillation |  | 201 (31.6) |  | 60 (29.3) |  | 38 (30.6) |  |  | 91 (34.1) |  | 12 (29.3) |  |
| Pacemaker rhythm |  | 109 (17.1) |  | 38 (18.5) |  | 21 (16.9) |  |  | 42 (15.7) |  | 8 (19.5) |  |
| Heart rate at baseline ┼ | 636 | 73 (65 - 83) | 204 | 72 (63 - 77) | 124 | 69.7 (62 - 76) | p=  0.12 | 267 | 79.1 (68 - 88) | 41 | 78 (66 - 88) | p=  0.80 |
| Ejection fraction (%) | 629 | 42.4 ± 14.3 | 202 | 43.2 ± 13.0 | 124 | 42.6 ± 14.2 | p=  0.69 | 263 | 41.3 ± 15.3 | 40 | 44.6 ± 13.7 | p=  0.19 |
| BMI | 604 | 27.4 ± 5.5 | 193 | 27.7 ± 5.5 | 123 | 27.8 ± 4.9 | p=  0.83 | 250 | 27.0 ± 5.7 | 38 | 27.0 ± 5.6 | p=  0.99 |
| **Lab** |  |  |  |  |  |  |  |  |  |  |  |  |
| NT-proBNP pmol/l ┼ | 161 | 195 (82-418) | 38 | 145 (44-250) | 22 | 111 (42-273) | p=  0.41 | 94 | 262 (109-528) | 7 | 165 (61-903) | p=  0.79 |
| Potassium <3.5 >5.0 mmol/l | 515 | 103 (16.2) | 159 | 19 (9.3) | 93 | 19 (15.3) | p=  0.09 | 232 | 55 (20.6) | 31 | 10 (24.4) | p=  0.30 |
| Sodium <135 > 145 mmol/l | 514 | 29 (4.6) | 159 | 6 (2.9) | 95 | 1 (0.8) | p=  0.14 | 229 | 20 (7.5) | 31 | 2 (4.9) | p=  0.67 |
| Creatinine >220 umol/l | 527 | 56 (8.8) | 163 | 4 (2.0) | 101 | 8 (6.5) | **p=**  **0.038** | 232 | 39 (14.6) | 31 | 5 (12.2) | p=  0.92 |
| Hemoglobin mmol/l | 196 | 7.9 ± 1.3 | 51 | 8.2 ± 1.1 | 42 | 8.1 ± 1.4 | p=  0.77 | 93 | 7.6 ± 1.2 | 10 | 7.8 ± 1.8 | p=  0.57 |
| Serum ferritin ug/l | 122 | 222.6 ± 227.7 | 34 | 172.7 ± 157.6 | 29 | 200.5 ± 158.6 | p=  0.49 | 52 | 287.2 ±289.1 | 7 | 76.9 ± 61.6 | **p<**  **0.001** |
| Transferrin saturation (%) ┼ | 120 | 20 (15-28) | 35 | 21 (15-30) | 27 | 21 (16-25) | p=  0.35 | 52 | 20 (15-28) | 6 | 15 (11-30) | p=  0.64 |
| **Medication** |  |  |  |  |  |  |  |  |  |  |  |  |
| Beta blocker | 637 | 513 (80.5) | 205 | 161 (78.5) | 124 | 110 (88.7) | **p=**  **0.013** | 267 | 210 (78.7) | 41 | 32 (78.0) | p=  0.93 |
| ACE inhibitor | 637 | 319 (50.1) | 205 | 107 (52.2) | 124 | 63 (50.8) | p=  0.81 | 267 | 131 (49.1) | 41 | 18 (43.9) | p=  0.54 |
| AT2 antagonist | 637 | 188 (29.5) | 205 | 64 (31.2) | 124 | 36 (29.0) | p=  0.68 | 267 | 75 (28.1) | 41 | 13 (31.7) | p=  0.63 |
| Diuretics | 637 | 506 (79.4) | 205 | 158 (77.1) | 124 | 99 (79.8) | p=  0.56 | 267 | 220 (82.4) | 41 | 29 (70.7) | p=  0.13 |
| Lanoxin | 637 | 122 (19.2) | 205 | 37 (18.0) | 124 | 25 (20.2) | p=  0.64 | 267 | 53 (19.9) | 41 | 7 (17.1) | p=  0.68 |
| **Device** |  |  |  |  |  |  |  |  |  |  |  |  |
| Pacemaker | 637 | 269 (42.2) | 205 | 87 (42.4) | 124 | 65 (52.4) | p=  0.08 | 267 | 106 (39.7) | 41 | 11 (26.8) | p=  0.10 |
| ICD | 637 | 157 (24.6) | 205 | 55 (26.8) | 124 | 40 (32.3) | p=  0.29 | 267 | 59 (22.1) | 41 | 3 (7.3) | **p=**  **0.003** |
| **Telemonitoring** | 637 | 69 (10.8) | 205 | 15 (7.3) | 124 | 13 (10.5) | p= 0.32 | 267 | 35 (13.1) | 41 | 6 (14.6) | p= 0.79 |
| **Comorbidities at baseline** |  |  |  |  |  |  |  |  |  |  |  |  |
| Hypertension | 637 | 260 (40.8) | 205 | 82 (40.0) | 124 | 46 (37.1) | p=  0.60 | 267 | 115 (43.1) | 41 | 17 (41.5) | p=  0.85 |
| COPD | 637 | 95 (14.9) | 205 | 29 (14.1) | 124 | 18 (14.5) | p=  0.93 | 267 | 43 (16.1) | 41 | 5 (12.2) | p=  0.52 |
| Diabetes mellitus | 637 | 168 (26.4) | 205 | 50 (24.4) | 124 | 29 (23.4) | p=  0.84 | 267 | 79 (29.6) | 41 | 10 (24.4) | p=  0.49 |
| Aortic valve stenosis | 637 | 69 (10.8) | 205 | 13 (6.3) | 124 | 15 (12.1) | p=  0.070 | 267 | 35 (13.1) | 41 | 6 (14.6) | p=  0.79 |
| Myocardial infarction | 637 | 191 (30.0) | 205 | 59 (28.8) | 124 | 39 (31.5) | p=  0.61 | 267 | 83 (31.1) | 41 | 10 (24.4) | p=  0.39 |
| CABG | 637 | 106 (16.6) | 205 | 30 (14.6) | 124 | 22 (17.7) | p=  0.46 | 267 | 47 (17.6) | 41 | 7 (17.1) | p=  0.93 |
| CVA | 637 | 48 (7.5) | 205 | 13 (6.3) | 124 | 10 (8.1) | p=  0.57 | 267 | 23 (8.6) | 41 | 2 (4.9) | p=  0.42 |

^Values are presented as number (%) or mean ±SD; ┼ = median (IQR 25-75); ACE=angiotensin-converting-enzyme; ATII=Angiotensin II; BMI= Body Mass Index; CABG=Coronary Artery Bypass Grafting; CVA=Cerebro Vascular Accident; Device= Pacemaker, ICD; MIS-HF=Maastricht Instability Score – Heart failure; n=number; NYHA=New York Heart Association Functional Classification; NTproBNP= N-terminal pro-brain natriuretic peptide.^
